# The Economic Impact of Lower Protein Infant Formula for the Children of Overweight and Obese Mothers

**DOI:** 10.3390/nu8010018

**Published:** 2016-01-02

**Authors:** Kevin Marsh, Jörgen Möller, Hasan Basarir, Panagiotis Orfanos, Patrick Detzel

**Affiliations:** 1 Evidera, Metro Building, 1 Butterwick, London W6 8DL, UK; Kevin.Marsh@evidera.com (K.M.); Jorgen.Moller@evidera.com (J.M.); 2 Roche, Konzern-Hauptsitz, Grenzacherstrasse 124, CH-4070 Basel, Switzerland; PanagiotisOrfanos@yahoo.gr; 3 Nestlé Research Center, 1000 Lausanne 26, Vaud, Switzerland; Patrick.Detzel@rdls.nestle.com

**Keywords:** obesity, infant formula, cost-effectiveness analysis

## Abstract

The global prevalence of obesity is rising rapidly, highlighting the importance of understanding risk factors related to the condition. Childhood obesity, which has itself become increasingly prevalent, is an important predictor of adulthood obesity. Studies suggest that the protein content consumed in infanthood is an important predictor of weight gain in childhood, which may contribute to higher body mass index (BMI). For instance, there is evidence that a lower protein infant formula (lpIF) for infants of overweight or obese mothers can offer advantages over currently-used infant formulas with regard to preventing excessive weight gain. The current study used health economic modelling to predict the long-term clinical and economic outcomes in Mexico associated with lpIF compared to a currently-used formula. A discrete event simulation was constructed to extrapolate the outcomes of trials on the use of formula in infanthood to changes in lifetime BMI, the health outcomes due to the changes in BMI and the healthcare system costs, productivity and quality of life impact associated with these outcomes. The model predicts that individuals who receive lpIF in infancy go on to have lower BMI levels throughout their lives, are less likely to be obese or develop obesity-related disease, live longer, incur fewer health system costs and have improved productivity. Simulation-based economic modelling suggests that the benefits seen in the short term, with the use of lpIF over a currently-used formula, could translate into considerable health and economic benefits in the long term. Modelling over such long timeframes is inevitably subject to uncertainty. Further research should be undertaken to improve the certainty of the model.

## 1. Introduction

The global prevalence of obesity has doubled in the last 30 years, with World Health Organization (WHO) estimates at approximately 10% in men and 14% in women [1]. This increase has compounded an already considerable public health burden, given the wide range of diseases related to being overweight [2], with obesity accounting for almost 7% of total healthcare costs globally [3].

This heavy disease toll makes it crucial to understand risk factors related to obesity. Among these is childhood obesity, an important predictor of adulthood obesity that has itself become increasingly prevalent. According to WHO, the annual rate of increase in the prevalence of childhood obesity is 10-times that of the 1970s [4]. Specifically, the global prevalence of overweight and obese children rose from 4.2% in 1990 to 6.7% in 2010 [5]. These epidemiological changes have long-term implications. A review [6] reported that overweight and obese children aged two to 17 years were over twice as likely as non-obese children to become overweight adults. Similarly, another review of longitudinal studies [7] found that individuals who were overweight or obese during infancy were more likely to develop obesity in later childhood, adolescence and adulthood. In addition, a meta-analysis of individual-level data from 10 cohort studies conducted in Finland, France, Seychelles, Sweden, the United Kingdom (U.K.) and the United States (U.S.) also suggested a strong positive association between infant weight gain and obesity, particularly childhood obesity [8].

This clear link between obesity in childhood and adulthood in turn invites questions about what influences the development of the condition so early in life. A key focus of interest and research in this area is the possibility that obesity may be programmed by changes in the expression of certain key genes (without the alteration of the genetic sequence) as a result of unfavourable environmental factors in utero and the postnatal period [9,10]. These so-called epigenetic changes are in keeping with knowledge that the pre-natal phase is crucial in infant growth and evidence that maternal weight is a key risk factor [10], perhaps by contributing to unfavourable nutrition of the foetus [9]. Specifically, maternal obesity has been linked to increased risk of obesity for the offspring later in life [11]. Furthermore, a systematic review of prospective observational studies that followed children from birth for at least two years found that significant risk factors for being overweight in childhood included the maternal overweight factor [12].

The pre-natal influence of maternal weight in programming obesity could be compounded by nutrition in the early post-natal phase, which has also been proposed as an important epigenetic influence [9,11]. Of relevance, breast-fed children are less likely than those fed on formula to experience rapid weight gain in infancy and subsequent obesity in childhood or adulthood [13,14]. The lower protein content of breast milk appears to contribute to this benefit [11]. The longer term impacts of consuming lower protein breast milk are achieved by impacting underlying metabolic programming, which in turn impacts the long-term risk of developing obesity [13,15,16].

The protein content of breast milk falls from up to 2.09 g/100 kcal in the first month after birth to around 1.28 g/100 kcal at three to four months and around 1.24 g/100 kcal by nine to 12 months [13]. By comparison, the lower regulatory limit for protein content of formula milk for children aged 0 to 12 months is 1.8 g/100 kcal in both the European Union (EU) and the U.S., with the actual protein content in formulas typically exceeding this level [13]. The differences in the content of breast milk and formula raised the possibility that re-adjusting the content of infant formula might help prevent rapid weight gain and obesity in infancy. 

This has now been demonstrated in a trial in Chile, in which infants whose mothers were overweight and who were predominantly formula-fed by three months were randomised either to lower protein infant formula (lpIF), low caloric density and probiotics included or to a currently-used formula [13]. Those fed the lpIF had gained less weight at 6, 12 and 24 months [13]. This result is supported by a multicentre European study where 1138 formula-fed infants were randomly assigned to receive cow milk-based infant and follow-on formula with lower or higher protein contents for a year. The lower protein intake was associated with lower weight in the first two years of life [17]. Other studies demonstrate that the positive effect of low protein intake is persevered over time, especially in children that are genetically predisposed to become obese**.** Once such study demonstrated the effect of low-protein infant formula on outcomes at three and five years of age [18]. These findings complement those of the Childhood Obesity Project, where infants randomised to receive a currently-used formula during the first year of life had a significantly higher body mass index (BMI) and a 2.4-times higher risk of being obese at six years of age than those given lower protein content formula [19].

The findings of the trial of lpIF could have major implications, particularly for countries with a significant prevalence of both maternal obesity and formula feeding. One such setting is Mexico. A review by the Organisation for Economic Co-operation and Development (OECD) reported that, in 2012, 37.5% of women in Mexico were obese, a rate exceeding that in all of the other countries considered (including the U.S., various EU countries, Australia and Canada) [20]. This prevalence exceeds even the very high figure for Chile (30.7%), the setting for the trial of lpIF. Furthermore, a survey of infant feeding practices in Mexico between 1999 and 2006 suggested trends largely towards lower rates of breast feeding, especially among vulnerable groups, such as indigenous people [21]. These findings suggest that preferential use of the lpIF over other formulas where mothers have stopped breastfeeding may confer significant benefits to their children, in Mexico and other settings.

Against this background, the current study used health economic modelling to investigate whether the benefits observed in the lpIF trial over a short period would translate into long-term clinical and economic advantages for the lpIF formula compared to a currently-used formula in Mexico.

## 2. Methods

### 2.1. Modelling Approach

Although cohort-level Markov models are the most commonly-used technique for pharmacoeconomic analysis [22], the lpIF model was implemented as a discrete event simulation (DES). This method was chosen as it allows a detailed and transparent simulation of population heterogeneity and competing health risks [23]. That is, DES enables the clinical history of individuals to be taken into account, which is important in obesity, and allows such individuals to be in multiple health states at any given time.

The model was applied to evaluate two alternatives for infant feeding:

- lpIF, which has low protein content and caloric density (1.65 g/100 kcal, 62.8 kcal/dL) and also contains probiotics.
- A currently-used formula with high protein content and caloric density (2.63 g/100 kcal, 65.6 kcal/dL).

**Figure 1:**
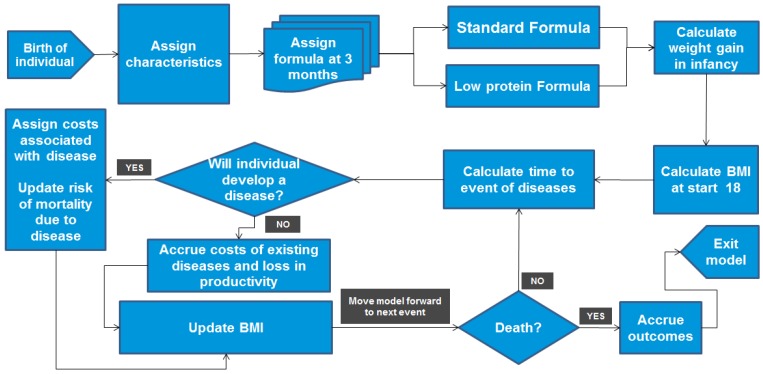
Flow diagram of the model; BMI, body mass index.

The model was designed to predict and allow comparison of the effects of the two formulas over the lifetime of individuals born to overweight (BMI 25 to 30 kg/m^2^) or obese (BMI ≥ 30 kg/m^2^) mothers in Mexico, by estimating lifetime BMI and the impact of BMI on health outcomes, health costs, quality of life and productivity. The model simulated 10,000 new-born infants who were assigned a set of individual characteristics, such as gender, maternal weight, BMI, birth weight and birth length, reflecting the Mexican population. Each “virtual” infant was then cloned, and one of each resulting pair was allocated to the lpIF group and the other to the current formula group. Each individual was modelled separately from birth to death, carrying their own unique characteristics and trajectories. The health outcomes and the costs were accumulated over time and discounted at 3.5%. The model was designed using the Arena^®^ software package (Rockwell Automation, Wexford, PA, USA) with a user interface in Excel^®^ (Microsoft, Redmond, WA, USA). Figure 1 illustrates the model structure and flow.

### 2.2. Model Inputs

Characteristics were assigned to individuals in the model based on the descriptive statistics of the Mexican population summarised in Table 1. These characteristics were selected as they were required to estimate the risk functions employed in the model.

**Table 1:** Individual characteristics.

Parameter	Mean Value	Standard Error	Source
Gender of new-borns (% male)	52.0%	-	[24]
Mean birth weight in Mexico (in grams)	3 202	472	
Mean birth height * in Mexico (in cm)	50.3	2.7	
Mean mother BMI in Mexico (kg/m^2^)	26.2	4.2	
Gestational age (weeks)	39.1	1.7	
Mean mother height (in cm)	155.4	5.7	
Head circumference (in cm)	34.3	1.8	
Maternal socioeconomic status (medium to low) **	59.5%	-	
% of mothers smoking ***	10.70%	-	
Race (% Caucasian)	87.6%	32.3% ****	[13]
Race (% Hispanic non-white)	12.4%	-	
Education (<4 years)	0.9%	-	
Education (4 to 8 years)	6.6%	-	
Education (8 to 9 years)	10.7%	-	
Education (≥10 years)	81.8%	-	
Family diabetes history (parent or sibling had diabetes)	29.5%	1.5% *****	[25]
Cholesterol/HDL-C ratio	Age and gender specific; see Table S22.		
Fasting glucose level (mg/dL)			
SBP level (mm Hg)			
HDL level (mg/dL)			
Smoking status			

HDL-C, high-density lipoprotein cholesterol; SBP, systolic blood pressure. * Due to the need to be consistent in the equations, “height” has been used even when referring to the “birth length”. ** Based on the fact that 59.5% of mothers have high school education or above, but overall, they are all “of medium to low socioeconomic status”. For the needs of the Ekelund Equation (Equation (S1)), it is assumed that 59.5% are skilled workers (6+ years after school) and the rest are unskilled (no education after school). *** Maternal smoking status is based on the percentage of female smokers in Mexico between the ages of 18 to 29. **** Standard deviation calculated from the lower protein infant formula (lpIF) trial. ***** The standard error is calculated based on the assumption that this variable is normally distributed with 95% of the area within 1.96 standard deviations of the mean.

### 2.3. BMI Trajectory

The trajectory of the individuals’ BMI over their lifetimes was modelled in four phases, reflecting the best data available for the following ages.

#### 2.3.1. BMI at Age 2 Years

BMI at 2 years old was estimated by using functions generated from an analysis of data from the trial of lpIF, a double-blind, randomised study of infants of overweight mothers (BMI > 25 kg/m^2^) [13]. A sample of 330 infants was screened, of whom 305 were enrolled, at either birth (*n* = 302) or between 5 and 31 days of age (*n* = 3). At 3 months of age, infants who were predominantly breast fed (*i.e.*, receiving no more than one formula feed per day) were assigned to a breast feeding reference group (*n* = 76). Predominantly formula-fed infants were randomly assigned to one of the study formulas: lpIF (*n* = 86) or a currently-used formula (*n* = 86). These formulas were used until the infants were12 months of age; height and weight were measured at 12 and 24 months.

This study was used to measure weight and height at 2 years, rather than relying on longer term trials [19], as it was more relevant. Specifically, it targeted the infants of overweight/obese mothers; the comparator was routine formula used in standard of care, and the study population was more similar to that in Mexico, the focus of the model. Furthermore, the team had access to the data, allowing functions to be generated.

BMI at 2 years of age was calculated from individual height and weight values, which were derived by developing mixed regression models with a random intercept (tools that offer a flexible and powerful approach to analysing repeated-measures data [26]. Relevant Equations (S-E1) to (S-E3) and Tables (S1 and S2) are reported in the Supplementary Information (SI).

#### 2.3.2. BMI at Age 17 Years

Table 2 reports the results of the analyses undertaken to estimate BMI at 17 years. This estimation was based on a function derived from an analysis of the Stockholm Weight Development Study (SWEDES) [27,28] that predicted BMI at age 17 years, based on BMI at 2 years and several other individual characteristics, adjusted to reflect the results of a more comprehensive meta-analysis based on U.S. data [29]. A scenario analysis was conducted to assess the impact of this adjustment. The relevant Equation (S-E4) and further information are provided in the SI.

**Table 2:** Regression model of BMI at age 17 [8] (Ekelund analysis *).

Parameter	Mean	Standard Error
Intercept	10.779	4.356
Weight gain in infancy at 24 months	1.788 **	0.171
Birth weight (kg)	1.761	0.426
Gender status (Female)	1.089	0.325
Gestational age (weeks)	0.106	0.114
Maternal low-medium socioeconomic status	−0.201	0.171
Maternal BMI (kg/m^2^)	0	0.042

BMI, body mass index. * These analyses were conducted by Dr. Ekelund, in addition to the analyses in his published study [30]. ** The adjustment of the Ekelund equation to match Druet is done by modifying the “weight gain in infancy” parameter from the original 1.090 to the above 1.788.

#### 2.3.3. BMI at Age 18 Years and Higher

Based on Østbye *et al.* (2011) [31], BMI between the ages of 18 and 48 years was predicted by means of BMI at 18 years and four different trajectory categories (Equation (S-E5) and Table S4 in the SI). BMI after 49 years of age was estimated based on BMI data from Mexico (Equation (S-E6) and Table S5 in the SI). A polynomial equation was fitted to WHO data on the cross-sectional mean BMI, by gender, for 10-year age groups between 49 and 79 years and the mean BMI between 80 and 100 years [32].

### 2.4. Disease Risks

The diseases experienced by individuals as a consequence of high BMI were assumed to occur only after the age of 18 years. After that age, several disease-specific risk functions were applied to predict the time until an individual would experience a primary event, specifically diabetes, myocardial infarction (MI), stroke or angina. At this point, the individuals’ characteristics were updated to reflect their updated disease history, age and BMI, and a new set of risk functions was applied to determine the timing of secondary events.

#### 2.4.1. Primary Events

The risk of diabetes was calculated using the San Antonio Equation [33]. The risk for chronic heart disease (CHD) and stroke was calculated using the Framingham equation [34]. A probability based on data from D’Agostino *et al.* (2000) [35] classified the initial CHD event as angina or MI [35]. Relevant equations and tables for primary events are Equations (S-E7) to (S-E9) and Tables S6 to S9 in the SI.

#### 2.4.2. Secondary Events

The risk of a secondary event depended on both the primary event experienced and the time period after the event, with probabilities of events being different during a 3-month acute phase after each event and a subsequent post-acute phase (Table S10 in the SI). The risk of developing a secondary cardiovascular event (MI or stroke) based on a 3-monthly cycle was estimated using published literature [36,37,38,39,40].

### 2.5. Mortality

Mortality was estimated using data from two sources. The risk of mortality as a result of death not attributable to any of the modelled disease events was derived separately for female and male individuals by fitting a Gompertz function piece-wise to the different age brackets in the all-cause mortality life tables for Mexico [41,42], having adjusted for the disease-specific mortality below (Table S11 in the SI). The risk of disease-specific mortality (primary and secondary) was calculated following MI and stroke events where the patient was exposed to the acute risk in the first 3 months after the event and then exposed to the post-acute risk, again with data being derived separately for females and males (Tables S12 to S14 in the SI). For angina, the risk was assumed to be constant after the event.

### 2.6. Healthcare Costs

Health system costs attributable to the health risks were estimated in Mexican pesos (MXN) (Table S15 in the SI). Costs were inflated to 2014 values using the national Mexican price index [43]. As the maintenance costs of chronic angina were not available for Mexico, they were estimated by deducting the costs for intensive care and surgical treatment from the annual cost for ischaemic heart disease. Costs for stroke and MI (fatal or non-fatal) in the acute phase are one-off costs applied once after the occurrence of the event. Costs for non-fatal stroke or MI in the post-acute phase were applied on a daily basis following the end of the acute phase. The costs for angina and diabetes were applied on a daily basis from the occurrence of the event to death.

### 2.7. Health-Related Quality-of-Life Impacts

Utility is assumed to be equal to 1 prior to the age of 18 years. After the age of 18 years, utility decrements were applied based on an individual’s age, BMI and disease history (Table S16 in the SI). 

### 2.8. Productivity Loss

Productivity losses were calculated using the capital approach and accounted for in two ways. Firstly, for a child up to the age of 12 years, a higher BMI was assumed to be associated with a greater chance of absenteeism from school. For the 20.7% where both parents work or a single parent works, this absenteeism consequently influenced the parents’ ability to work. Secondly, the development of BMI-related diseases was assumed to be associated with workplace absenteeism in adults. The productivity impacts of MI, stroke, angina and diabetes were based on employment rate by age range, gender and the following assumptions: retirement age of 65 years and individuals working for 257 days every year. Additional details are provided in Tables S17 to S19 in the SI.

## 3. Results 

### 3.1. Base-Case Results

#### 3.1.1. Clinical Outcomes

The model predicts that individuals who receive lpIF in infancy have lower BMI levels throughout their lives (Table 3). Specifically, over their lifetimes, these people are 10.5% less likely to be obese than are those fed a currently-used formula. Furthermore, lpIF-fed infants are 2.2% to 3.3% less likely than those given a currently-used formula to experience obesity-related diseases.

**Table 3:** Base-case clinical outcomes.

Clinical Outcomes	lpIF	Currently Used Formula	Absolute Difference	Relative Difference
Average BMI (kg/m^2^) outcomes estimated by the lpIF model per individual over time (undiscounted)				
Average BMI at 18 years old	24.8	25.8	−1.0	−3.9%
Average BMI at 30 years old	26.6	27.7	−1.1	−4.1%
Average BMI at 45 years old	28.1	29.0	−1.0	−3.4%
Average BMI at 60 years old	29.2	30.1	−0.9	−3.0%
Average lifetime BMI	27.3	28.2	−1.0	−3.5%
% of population becoming obese (BMI ≥ 30)	15.5%	17.1%	−1.6%	−10.5%
Years in obese state	2.4	2.6	−0.2	−8.1%
Probability of experiencing clinical events				
Diabetes	14.4%	14.8%	−0.4%	−2.9%
Angina	8.3%	8.6%	−0.3%	−3.3%
Myocardial infarction	3.2%	3.3%	−0.1%	−2.2%
Stroke	0.267%	0.274%	−0.007%	−2.9%

BMI, body mass index, lpIF, lower protein infant formula.

#### 3.1.2. Economic Outcomes

The reduced risk of disease in individuals fed with lpIF translates into lifetime economic benefits of MXN 984 per individual (Table 4). This saving occurs because lpIF-fed infants incur lower health system costs (reduced by MXN 260 or 24%) than do those given the currently-used formula. A reduction in both BMI and disease risk from the use of lpIF also translates into better productivity, with lpIF-fed infants incurring MXN 724 (74%) less in productivity losses than those fed with currently-used formula.

Infants fed with lpIF live 2.7 days longer than those fed with the currently-used formula, and their health-related quality of life (HRQL) improves by the equivalent of an additional 14.6 days in perfect health. However, once discounted, these gains diminished to 0.2 days (0.001 years) in life expectancy and 4.3 days in perfect health (0.01 quality-adjusted life-years (QALYs)) (Table 4).

**Table 4:** Base-case economic outcomes.

Economic Outcomes	lpIF	Currently Used Formula	Absolute Difference	Relative Difference
HRQL (discounted)				
Life years	26.098	26.097	0.001	0.002%
QALYs	24.76	24.75	0.01	0.05%
Direct health costs per person (2014 MXN, discounted)				
Diabetes	4394	4569	−175	−4.0%
Angina	721	751	−30	−4.2%
Myocardial infarction	32	34	−1	−3.4%
Stroke	1568	1622	−54	−3.5%
Total	6715	6975	−260	−3.9%

HRQL, health-related quality of life; QALY, quality-adjusted life year; lpIF, lower-protein infant formula; MXN, Mexican pesos.

#### 3.1.3. Sensitivity Analyses

The probability of lpIF being cost effective was determined using two methods. One threshold (MXN 73262 per QALY) was derived using the WHO method; this assumes that an intervention with an incremental cost-effectiveness ratio (ICER) below the GDP per capita of a country is very cost effective. The other threshold (MXN 219696 per QALY) was derived using an alternative method also by WHO, which assumes that an intervention with an ICER below three times the GDP per capita of a country is cost effective. The probability of lpIF being cost effective was 60.6% and 58.2%, respectively. The details of the probabilistic sensitivity analysis (PSA) outcomes are provided in Figure S1 and Table S20 in the SI.

### 3.2. Scenario Analyses

Table 5 reports the outcomes of the five scenario analyses conducted to explore the robustness of the results to some of the key assumptions made in the model.

Discounting: Guidelines [44] suggest that a lower discount rate may be used in analyses over prolonged periods of time. To explore the impact of discounting, our model was run without discounting (*i.e.*, with a 0% discount rate), rather than the 3.5% discount rate used in the base case. Unsurprisingly, the cost savings increased significantly, to MXN 7241.

Trial population: The base case draws on Mexican population data, which reflects the whole population, rather than the characteristics of infants of overweight or obese mothers specifically. To test the impact of this, the characteristics of the sample captured in the lpIF trial were used instead. In this instance, the predicted cost savings were only marginally impacted.

Period observed in the trial: The base-case analysis uses 24-month trial data to predict BMI in infanthood. A scenario was run using the 12-month trial data and then extrapolating from 12 months to 17 years using alternative predictive functions (Table S3 in the SI). This scenario reduced the cost savings to MXN 265.

Valuing productivity losses: The base-case analysis employs the capital approach to value productivity losses. A scenario was run using the friction method to value productivity losses, which caused the cost savings to increase to MXN 1456.

Adjustment factor in predicting for BMI at age 17 years: An adjustment factor has been used to avoid a potential underestimation of the BMI prediction at age 17 years (see Table 5). A scenario was run without using this adjustment factor. This reduced the cost savings to MXN 657.

**Table 5:** Results of the scenario analyses.

Scenario	Costs Absolute Difference, 2014 MXN (lpIF *vs.* Currently-Used Formula)	Costs Relative Difference (lpIF *vs.* Currently-Used Formula)
Base case	−984	−4.05%
Undiscounted outcomes	−7241	−3.95%
Individual characteristics based on the lpIF Chilean trial population	−1034	−4.36%
Trial data used to observe impact over 12 months	−265	−1.04%
Valuing productivity losses using the friction approach	−1456	−1.25%
Ekelund equations at age 17 without the adjustment factor	−657	−2.79%

lpIF, lower-protein infant formula; MXN, Mexican pesos.

### 3.3. Validation

Modelling outcomes over the long timeframes required to estimate the value of lpIF introduces significant uncertainty. To assess the validity of the predictions of the model, its results specified for the current situation in Mexico (*i.e.*, using individual characteristics based on descriptive statistics for the Mexican population and assuming the use of the currently-used formula) were compared to the observed outcomes for the Mexican population (Table S21 in the SI).

This analysis demonstrated that the model accurately predicts the current life expectancy in Mexico (predicted: 77.5 years; observed: 77 years). However, the model underestimates the average BMI (predicted: 28.2 kg/m^2^; observed: 31.3 kg/m^2^) and the likelihood of developing diabetes, MI, angina and the probability of being obese, but slightly overestimates the probability of experiencing stroke (predicted risk: 0.27%; observed risk: 0.21%).

## 4. Discussion

If the current worldwide childhood obesity epidemic carries on into adulthood, the considerable demand this condition already places on healthcare services will increase substantially [4]. This emphasises the importance of measures that aim to stem the increasing occurrence of obesity, and early childhood may be the best opportunity to employ these [4]. The findings of the lpIF trial indicate a potential way of limiting the tendency to childhood obesity in a setting such as Mexico, where the risk of this condition is heightened by a high prevalence of maternal obesity and an increase in the protein intake at infancy and early childhood. Through DES modelling, our study shows that, for the children of overweight and obese mothers, reduction in weight gain through the use of the lpIF rather than a currently-used formula would result in a lower BMI in adulthood. This, in turn, would help prevent obesity-related diseases, bring about healthcare cost savings and improvements in productivity.

In considering the results of our analysis, it is important to note that the protein content of the lpIF is below the lower limit set for formula milk by regulatory authorities in the EU and U.S. This raises questions about whether restrictions on the availability of lower protein formulas remains appropriate, given the rising levels of maternal obesity and the mounting evidence of the relationships between higher protein formula intake, rapid weight gain and obesity in early life. It might also raise questions about the safety of the product. However, trial data demonstrate that there are no issues regarding the safety of the product despite the protein level being below the limit set by the regulatory authorities [45]. 

As far as we are aware, this is the first study to use DES modelling to predict the long term clinical and health economic consequences of using lower protein content formula, such as the lpIF. Building such a model is, however, subject to various challenges. Our analysis predicts outcomes for a population in Mexico, but relies on efficacy data from the lpIF trial, which was conducted in Chile. This concern was partly overcome by the use of an individual-level model, which allowed the Mexican population characteristics to be run through a function developed from the Chilean trial data. In addition, volumetric intake was controlled in both arms of the trial. Further work is required to evaluate whether lower protein formula is associated with high volumetric intake in a real-world setting. 

Other limitations of our study relate primarily to the challenges in constructing the individual-level model and the uncertainty associated with such long-term modelling. This is evident in the spread of outcomes observed in the PSA results, which indicate that in only 58.2% and 60.6% of model iterations (depending on the threshold used) is lpIF considered cost effective. One of the contributors to this uncertainty is that the modelling of BMI from infanthood through to death required the use of four functions derived from separate sources, each of which may be critiqued as to its suitability for such an application. 

For instance, the function used to estimate BMI at age 17 years is derived from an analysis of Swedish data, and published meta-analyses suggest this source underestimated the impact of infant weight gain on adult BMI. Therefore, the functions derived from the Swedish data in our study were adjusted to reflect this underestimate. Further work should be undertaken to generate risk functions based on data sources more representative of the Mexican population. 

The BMI trajectory from ages 18 to 49 years also has limitations. It offers advantages by distinguishing a rate of change in BMI for four groups defined by the BMI level at 18 years old. However, while this improves on current modelling approaches, which tend to assume a constant rate of weight gain per year over time [46], it would be even more accurate if functions were developed to predict the rate of BMI change based on the individual level BMI at 18 years old. In addition, the BMI trajectory after 49 years of age is based on cross-sectional data of the current Mexican population, evidence that cannot therefore reflect different BMI trajectories across the population. The data are further limited for our purposes in that only some of sampled individuals would have been the infants of overweight or obese mothers, and all of them would have lived in a very different Mexico from the one in which our target population is growing up. By comparison, retrospective individual or grouped data would have allowed for different BMI trajectories, as modelled for the ages of 18 to 49 years. These data limitations mean that the gains in BMI that would be observed in reality in the target group during this age band have possibly been underestimated, which would lead to the underestimation by our model of the impact of lpIF on BMI levels.

Finally, our model considers the impact of BMI on health outcomes only after the age of 18 years, as no rigorous evidence was identified on the impact of BMI on health outcomes for the younger age groups. However, increasingly, findings of observational studies seem to point to early deleterious health consequences of being overweight and obesity [47]. In addition, the model considers only the key cardiovascular impacts of BMI. Further work is therefore needed to explore the BMI-related complications for younger age groups and to extend the model to other associated diseases, including osteoarthritis and colon cancer: two common non-communicable diseases that have clear associations with being overweight and obesity [48,49].

## 5. Conclusions

An economic model based on a DES suggests that the short-term benefits of using lpIF, preventing rapid weight gain in infancy, could translate into considerable health and economic benefits in the long term, including a 10.5% reduction in the likelihood of developing obesity and a 3.9% reduction in direct health costs.

## Supplementary Materials

Supplementary materials can be accessed at: http://www.mdpi.com/2072-6643/8/1/0018/s1.
