# Supplementary material for: The Economic Impact of Lower Protein Infant Formula for the Children of Overweight and Obese Mothers
Source: Nutrients. 2016 Jan 2;8(1):18. doi: 10.3390/nu8010018 (PMC4728632; doi:10.3390/nu8010018)
Supplement: Supplementary file 1 [file nutrients-08-00018-s001.pdf]

Supplementary Information: The Economic Impact of Lower Protein Infant Formula for the Children of Overweight and Obese Mothers

Kevin Marsh, Jörgen Möller, Hasan Basarir, Panagiotis Orfanos and Patrick Detzel

1. Methods

1.1. BMI Trajectory

1.1.1. BMI at 2 Years Old

The regression models for weight and height estimates are reported in Tables S1 and S2, respectively. The functions used for these estimations and the body mass index (BMI) calculation are reported in Equations (S-E1) to (S-E3), respectively.

Weight (kg) = Birth Weight + intercept + b1×(birth weight) + b2 × (birth height) + b3 × (female) + b4 × (Caucasian) + b5 × (mother BMI) + b6 × (education (4 to 8 years)) + b7 × (education (8 to 9 years)) + b8 × (education (+10 years)) + b9 × (mother is smoker) + b10 × (lpIF formula) +b11 × (age in months) + b12 × (age in months ^2) + b13 × (lpIF formula × age in months) + b14 × (lpIF formula × age in months ^2) + b15 × (female × age in months) + b16 × (female × age in months^2) + b17 × (female × lpIF formula × age in months) + b18 × (female × lpIF formula × age in months^2) (S-E1)

Height (cm) = BirthHeight + intercept + b1 × (square root of age in months) + b2(lpIF) + b3 × (square root of age in months × lpIF) + b4 × (Birth Height) + b5 × (female) + b6 × (mother height) + b7 × (head circumference) + NormalDist(0,√sigma1) + [square root of age in months × Normal.Dist(0,√sigma2)] + Normal.Dist(0,√sigma3) + 2 × Covariance{N.Dist(0,√sigma1),N.Dist(0,√sigma2)} (S-E2)

Table S1. Regression output for weight estimation at the age of 2 years.

Parameter	Mean	Standard Error
Intercept	−1.44494	1.4374
Birth weight	0.74178	0.13984
Birth height	0.05916	0.03225
Gender (Female)	−0.08303	0.10749
Race (Caucasian)	−0.12063	0.16784
Mother's BMI	0.00241	0.01439
Education (4 to 8 years) *	−0.2956	0.43465
Education (8 to 9 years) *	0.22165	0.42106
Education (+10 years) *	−0.2448	0.39849
Mother is smoker	0.17417	0.08865
lpIF formula	0.06003	0.1086
Age in months	0.79599	0.01751
Age in months ^2	−0.01681	0.00069
lpIF formula * age	−0.035	0.02371
lpIF formula * age^2	−0.00005	0.00096
Female * age in months	−0.06204	0.02359
Female * age in months ^2	0.00227	0.00094
Female and lpIF formula * age	0.06626	0.03115
Female and lpIF formula * age^2	−0.00103	0.0013

* Compared to the reference group: education (<4 years).

Table S2. Regression output for height estimation at the age of 2 years.

Parameter	Mean	Standard Error
Intercept	−2.824	3.1199
√Age (in months)	8.4893	0.0775
Formula (lpIF)	0.03166	0.286
Formula (lpIF)*[√Age(in months)]	−0.1146	0.1143
Birth height (cm)	−0.3894	0.04947
Gender status (Female)	−0.5712	0.1589
Mother's height (cm)	0.06574	0.01438
Head circumference at birth (cm)	0.2191	0.07691
Sigma 1 (1st correlation parameter for random effects model)	2.127	-
Sigma 2 (2nd correlation parameter for random effects model)	0.332	-
Sigma 3 (3rd correlation parameter for random effects model)	1.3545	-
Covariance of normal distribution (0, Sigma 1) and normal distribution(0, Sigma 2)	−0.5689	-

The model uses the functions reported in Tables S1 and S2 to estimate an individual's weight and height at age 2 years. Equation (S-E3) then uses these estimates to calculate BMI at age 2 years:

$$\text{BMI} = \text{Weight (kg)}/[\text{Height(m)}/100)]^2 \quad \text{(S-E3)}$$

1.1.2. BMI at 17 Years Old

The model uses the function summarised in Equation (S-E4) and Table S3 to predict an individuals' BMI at 17 years old.

$$\text{BMI (17 years)} = \alpha + \text{b1} \times \text{weight gain in infancy} + \text{b2} \times \text{birth weight} + \text{b3} \times \text{gender (female)} + \text{b4} \times \text{gestational age} + \text{b5} \times \text{maternal socioeconomic status} + \text{b6} \times \text{maternal BMI} \quad \text{(S-E4)}$$

The Stockholm Weight Development Study (SWEDES) study is based on a sample of 2342 mothers invited to participate between 1984 and 1985 and who were followed during and after their pregnancies. A total of 1423 of these mothers completed the study at the one-year follow-up, and 481 mothers and their children participated in the follow-up study (SWEDES) after 17 years.

A meta-analysis of 10 studies suggested that the analysis of SWEDES may underestimate the impact of weight gain in infancy on BMI at age 17 years [1]. To account for this underestimate, the coefficient on the weight gain variable in the original model was inflated by the difference between the odds ratio from the SWEDES cohort and that from the Philadelphia Blood Pressure Project (PBPP) cohort. The PBPP study was selected as it estimated the relationship between infant weight gain and BMI at 20 years old, the closest age of the studies included in the meta-analysis to that in the model. PBPP is a cohort of 300 African Americans born between 1962 and 1966 in the United States (U.S.) and followed up to 20 years of age.

Table S3. BMI at 17 years based on weight gain between birth and 12 months (Ekelund analysis *).

Coefficient	Value	Standard Error
Intercept	13.56	4.48
Weight gain in infancy	1.34 **	0.18
Birth weight (kg)	1.52	0.46
Gender status (female)	1.12	0.35
Gestational age (weeks)	0.07	0.12
Maternal socioeconomic status	−0.27	0.18
Maternal BMI (kg/m²)	−0.01	0.04

* These analyses were conducted by Dr. Ekelund, in addition to the analyses in his published study [33]. ** The adjustment of the Ekelund equation to match Druet *et al.* [1] is done by modifying the weight gain in infancy parameter from the original 0.82 to the above 1.34.

1.1.3. BMI at Age 18 Years and Higher

Individuals' BMI at 48 years old is predicted based on their weight at 17 years old. Østbye (2011) [2] generated a function (Equation (S-E5)) predicting BMI at age 48 years based on BMI at age 17 years.

$$\text{BMI} = (\text{BMI at age 17}) + \text{linear term} \times \text{age} + \text{quadratic term} \times (\text{age}^2) \quad \text{(S-E5)}$$

Østbye (2011) [2] specified Equation (S-E5) for four different subgroups, distinguished by their BMI at age 17 years old. The subgroups and the model parameters are reported in Table S4. In the discrete event simulation (DES), individuals are categorised into one of these subgroups based on their BMI at 17 years old, and the relevant function is selected to estimate the change in their BMI over time until the age of 48 years old.

Table S4. BMI classifications (kg/m^2) used in the estimation of BMI between the ages 18 and 48 years [2].

Subgroup [Range of BMI]	Linear Term	SE (Linear Term)	Quadratic Term	SE (Quadratic Term)
Normal weight [0, 20.82]	0.12	0.003	0	0
Overweight [20.83, 23.17]	0.31	0.013	−0.003	0.0004
Late adulthood [23.18, 26.64]	0.58	0.023	−0.008	0.0007
Early adulthood [26.65, 100]	1.05	0.068	−0.019	0.0024

1.1.4. BMI from 49 Years of Age Onwards

Individuals' BMI from age 49 years onwards is predicted based on a function derived from fitting a polynomial equation (Equation (S-E6)) to WHO data on the cross-sectional average BMI, by gender, for 10-year age groups between 49 and 79 years and the average BMI between 80 and 100 years [3].

$$\text{BMI} = (\text{BMI at age 49}) + \text{linear term} \times \text{age} + \text{quadratic term} \times (\text{age}^2) \quad \text{(S-E6)}$$

An individual's BMI at age 49 years is predicted by the model (see section BMI between the ages of 18 and 48 years above). The estimates of the linear and quadratic terms for the equation above are provided in Table S5.

Table S5. Linear and quadratic terms used in the projection of BMI for ages 49 years and above [4].

Gender	Linear Term	Quadratic Term
Males	0.0794	−0.0011
Females	0.0894	−0.0012

1.2. Disease Risks

1.2.1. Primary Events

Equation (S-E7) and Table S6 report the function used to determine the probability that an individual would experience diabetes over a period of 7.5 years.

$$\text{P (diabetes)} = 1/[1 + e \wedge (-1 \times (-13.415 + 0.028 \times \text{age} + 0.661 \times \text{sex} + 0.412 \times (\text{Mexican}) + 0.079 \times (\text{fasting glucose}) + 0.018 \times \text{SBP} - 0.039 \times \text{HDL} + 0.070 \times \text{BMI} + 0.481 \times (\text{prior family history of diabetes}))] \quad \text{(S-E7)}$$

If the individual is not expected to experience a disease-related event in the next 7.5 years, their risk would be re-assessed after this period for the following 7.5 years, and so on, until their death.

Table S6. San Antonio equation used to estimate the probability of diabetes risk [5].

Parameter	Mean	Standard Error *
Intercept	−13.42	0.68
Age (years)	0.03	0.00
Gender (female)	0.66	0.03
Race (Mexican American)	0.41	0.02
Fasting glucose level (mg/dL)	0.08	0.00
SBP level (mm Hg)	0.02	0.00
HDL level (mg/dL)	−0.04	0.00
BMI level (kg/m^2)	0.07	0.00
Family diabetes history (≥1 parent or sibling had diabetes)	0.48	0.02

* The standard error is calculated based on the assumption that this variable is normally distributed with 95% of the area within 1.96 standard deviations of the mean.

CHD Risk

Table S7 describes the function (Equation (S-E8)) used to estimate the probability of an initial CHD event.

$$P\,(CHD) = 1 - \exp\{-[(X\ days)\exp(-[14.9756 - 0.0159 \times BMI - 0.0571 \times age - 0.4959 \times (smoker) - 0.0070 \times SBP - 0.1432 \times cholesterol\ to\ HDL\ ratio - 0.3421 \times (diabetic) + 0.5139 \times ([female]\ ^\wedge\ (1/0.7303))\} \quad (S\text{-}E8)$$

Table S7. Framingham equation used to estimate the probability of initial CHD risk [6].

Parameter	Mean	Standard Error *
Intercept	14.98	0.76
BMI level (kg/m^2)	−0.02	0.00
Age (years)	−0.06	0.00
Smoking status (smoker)	−0.50	0.03
SBP level (mm Hg)	−0.01	0.00
Cholesterol/HDL-C ratio	−0.14	0.01
Diabetic status (diabetic)	−0.34	0.02
Gender (female)	0.51	0.03
Weibull lpIF	0.73	0.04

* The standard error is calculated based on the assumption that this variable is normally distributed with 95% of the area within 1.96 standard deviations of the mean.

Table S8 shows the probability that an initial CHD is either an MI, an angina or a cardiac death.

Table S8. Probability that an initial CHD event is angina or myocardial infarction (MI) * [7].

Gender	MI		Angina	
	Mean	Standard Error	Mean	Standard Error
Female	42%	2%	56%	3%
Male	53%	3%	41%	2%

* These do not add up to 100% since the remaining probability is associated with the initial CHD event being cardiac death.

Stroke Risk

Equation (S-E9) provides the function used to estimate the probability that an individual experiences a stroke.

$$P\ (\text{stroke}) = 1 - \exp\ \{-[(X\ \text{days})\ \exp(-[14.6574 - 0.0227 \times \text{BMI} - 0.0450 \times \text{age} - 0.2584 \times (\text{smoker}) - 0.007879 \times \text{SBP} - 0.0596 \times \text{cholesterol to HDL ratio}\ ]\ \hat{}\ 1/0.4978\} \quad \text{(S-E9)}$$

The estimates of the coefficients for the equation above are provided in Table S9.

Table S9. Framingham equation used to estimate the probabil0ity of initial stroke risk [6].

Parameter	Mean	Standard Error *
Intercept	14.66	0.75
BMI level (kg/m²)	−0.02	0.00
Age (years)	−0.05	0.00
Smoking status (smoker)	−0.26	0.01
SBP level (mm Hg)	−0.01	0.00
Cholesterol/HDL-C ratio	−0.06	0.00
Weibull lpIF	0.50	0.03

* The standard error is calculated based on the assumption that this variable is normally distributed with 95% of the area within 1.96 standard deviations of the mean.

1.2.2. Secondary Events

The sources used to estimate the risk of secondary events, contingent upon the nature of the primary event and the time since the primary event, are described in Table S10.

Table S10. The probability of secondary events after primary event, per three months.

Primary Event	Secondary Event	Phase	Mean	Standard Error	Source
MI	MI	Acute	2.12%	0.11%	1
		Post-acute	0.52%	0.03%	8
	Stroke	Acute	0.37%	0.02%	9
		Post-acute	0.11%	0.01%	8
Stroke	Stroke	Acute	1.46%	0.07%	10
		Post-acute	1.46%	0.02%	11
	MI	Acute	0.31%	0.02%	10
		Post-acute	0.31%	0.02%	11
Stroke and MI	Stroke	Post-acute	1.46%	0.07%	10
	MI	Post-acute	0.31%	0.02%	10
Angina	MI *	-	0.21%	0.01%	12
	Stroke *	-	0.15%	0.01%	12

* These probabilities are used in both acute and post-acute phases.

1.3. Mortality

There are two sources of mortality in the model. First, background mortality, which is assigned to each individual at birth, is the time of death provided the person does not die from any of the modelled disease events. Table S11 shows the functions used to estimate background mortality. These are generated by fitting a Gompertz function (parameters λ and γ) piece-wise to the different age brackets in the all-cause mortality life tables for Mexico [4].

Table S11. Background all-cause mortality functions.

Gender	Parameter	Age			
		Part 1	Part 2	Part 3	Part 4
Female	λ	−7.663730	−9.314051	−10.583913	−5.072112
	γ	−0.045055	0.073045	0.092736	0.037065
Male	λ	−4.496873	−8.184782	−7.455439	−9.413828
	γ	−1.596605	0.012823	0.047107	0.081203
Age Survival cut point: females †		16	72	95	110
Age Survival cut point: males †		2	14	64	105

† These age cut points define the different pieces of the piece-wise Gompertz function that was fitted to the background mortality hazard.

Second, experiencing disease events is associated with a mortality risk. The background mortality is adjusted for death associated with the cardiovascular events predicted separately in the model in order to avoid double counting. The data used to do this are reported in Table S12. Since diseases occur in the model only after the age of 18 years, background mortality was not adjusted prior to that age.

Table S12. Percent of deaths attributable to cardiovascular events.

Gender	Age (years)					Source
	18 to 24	25 to 34	35 to 44	45 to 64	65+	
Females	2.8%	4.6%	8.4%	15.9%	24.1%	[13]
Males	4.4%	6.4%	9.1%	13.1%	25.9%	

These disease-specific mortality risks comprises two elements. First, the probability that an initial CHD event is fatal is reported in Table S13.

Table S13. Proportion of initial CHD events that are cardiac death [7].

Gender	Mean	Standard Error
Female	2%	0.10%
Male	6%	0.31%

Second, the mortality rate subsequent to experiencing a disease event is reported in Table S14.

Table S14. Mortality rate following disease events (3-month probability).

Parameter	Mean	Standard Error	Source
MI in Acute	13.7%	0.70%	[8,14] *
Stroke in Acute	19.9%	1.02%	[10,14] **
MI in Post-acute	0.17%	0.01%	[8,14] *
Stroke in Post-acute	0.54%	0.03%	[10,14] ***
Stroke + MI in Post-acute	0.54%	0.03%	[10]
Angina	0.18%	0.01%	[12]

* Code "I63" is used "diag-09-10" worksheet of Hospital Episode Statistics (HES) (2010). ** Code "I21" is used "diag-09-10" worksheet of HES (2010). *** Code "I22" is used "diag-09-10" worksheet of HES (2010).

1.4. Healthcare Costs

Table S15 reports the data used to calculate the costs of diseases. Costs are inflated to 2014 values using the national Mexican price index [15].

Table S15. Direct medical costs of diseases (MXN, 2014).

Disease	Phase	Mean	Standard Error *	Source
Stroke	Acute (one-off cost after event)	44,080	2,249	[16]
	Post-acute (daily)	39.06	1.99	[17]
MI	Acute (one-off cost after event)	173,112	8,832	[16]
	Post-acute (daily)	44.69	2.28	[17]
Angina	All (per day)	181.38	9.25	[17]
Diabetes	All (per day)	37.24	1.90	[18]

* The standard error is calculated based on the assumption that this variable is normally distributed with 95% of the area within 1.96 standard deviations of the mean.

1.5. Health-Related Quality-of-Life Impacts

Table S16 reports the parameter values, dispersion parameters, descriptions and sources for the utility decrements.

Table S16. Utility decrements.

Parameters	Mean Decrement	Standard Error	Description	Source
Age	−0.0036	0.0002 *	Age-based annual utility decrement (applied starting from age 18)	[19]
BMI, non-diabetic	−0.0143	0.0008 *	Applied per unit increase in BMI for non-diabetic individual (applied starting from age 18)	[20]
BMI, diabetic	−0.0285	0.0015 *	Applied per unit increase in BMI, for diabetic individual (applied starting from age 18)	[20]
Acute MI	−0.0626	0.0132	Applied after the occurrence of the event	[19]
Acute stroke	−0.1171	0.0121	Applied after the occurrence of the event	
Post-acute MI	−0.0627	0.0131	Applied in post-acute phase (3 months following the event) until time of death	
Angina	−0.0854	0.0134	Applied until time of death	
Post-acute stroke	−0.0732	0.0244	Applied in post-acute phase (3 months following the event) until time of death	

* This value is calculated based on the assumption that this variable is normally distributed with 95% of the area within 1.96 standard deviations of the mean.

1.6. Productivity Impacts

Productivity impacts are incorporated into the model in two ways. First, before the age of 13 years, an individuals' BMI will impact their probability of missing school, with a knock-on impact on their parents' ability to attend work. Second, after the age of 18 years, employment status and productivity are impacted by the experience of health events.

1.6.1. School Absenteeism

Table S17 describes the function used to estimate the number of days individuals miss from school between the ages of 4 years and 12 years. Geier *et al.* (2007) [21] estimated the function from data collected from a cohort of 1069 fourth to sixth graders in the U.S. BMI was estimated by assuming a linear relationship between BMI at 2 years old and 17 years old.

The impact of school absence on productivity is estimated by assuming that a day off school would cause one parent to miss work for the 20.7% of children in Mexico for whom both parents work [22].

Table S17. Regression model of a day's absence from school [21].

Parameter	Mean	Standard Error
Intercept	2.22	3.86
Obese *	1.92	0.77
Overweight	1.04	0.86
Underweight	−1.41	2.21
Normal-weight	0	0
Black	−2.19	1.11
Asian	−7.5	1.22
Hispanic (Mexican American)	−1.68	1.19
Other	−2.36	1.86
White	0	0
Gender (Female)	−0.06	0.63
Age	1.1	0.32

* Obese here was defined as BMI for age ≥95th percentile.

1.6.2. Disease-Related Productivity Impacts

After the age of 18 years, for those individuals who are employed (Table S18), productivity loss is estimated based on days missed from work due to disease events. In a small proportion of instances, disease events will lead to individuals being permanently out of employment. In most instances, disease events are associated with a period off work. Table S19 summarises the data used to estimate these productivity impacts. Productivity losses are accrued until the individual retires at 65 years old [21] and assuming 257 working days per year.

For chronic conditions, such as diabetes and angina, a certain number of days is expected to be missed from work every year [23,24]. If, for example, diabetes causes 40 days off work per year, the patient is assumed to be off work 10 days at each quarter of the year.

If two disease events are experienced simultaneously, the highest number of days off work associated with these events is applied.

Table S18. Productivity impacts of obesity-related diseases.

Obesity-Related Diseases	Days off Work	Standard Error	Source	% Patients Permanently Disabled	Source
Myocardial infarction	168 per event	8.6	[25]	0.32%	[23]
Stroke	270 per event	13.8	[26]	0.28%	[24]
Diabetes	38 per year	1.9	[27]	0.00%	Assumption
Angina	2.0 per year	5.0	[28]	0.00%	Assumption

Table S19. Employment rate in Mexico by age and gender [29].

Age (Years)	Males (%)	Females (%)
18 to 24	55.6%	30.7%
25 to 34	90.1%	52.6%
35 to 44	92.6%	55.9%
45 to 54	90.3%	51.6%
55 to 64	76.6%	37.2%
65+	0.0%	0.0%

1.6.3. Valuing Productivity Impacts

The value of the productivity impact is estimated using the capital approach in the base case and with the friction approach in a scenario analysis. The capital approach assumes that an absent employee will never be replaced at work by another individual and is estimated using Equation (S-E10):

Productivity cost loss = total days of work loss × (elasticity of productivity × mean daily salary) (S-E10)

where the elasticity of productivity is 0.8, the proportion of the day during which the individual is actually productive [30] and the average daily salary is MXN 268.10 [31].

The friction approach assumes that absent employees will be replaced after a "friction" period, the time required to replace a person at work. If an individual is absent from work for a period less than the friction period, the friction method reduces to the capital method. If, however, the period of absence is greater than the friction period, the friction method estimates productivity costs using Equation (S-E11).

Productivity cost loss = (friction days × (elasticity of productivity × mean daily salary) + friction costs (S-E11)

where the friction period is 68.32 days (assumed to be the same in Mexico as in the U.K. [31]), and the friction cost (one-off cost of replacing an employee: vacancy cover, redundancy cost, recruitment and selection, training and induction cost) is MXN 26,266 [30].

2. Sensitivity Analysis for the Base Case Results

Figure S1 shows the outcome of the PSA. Each blue diamond point on the figure is the outcome of a model run, undertaken to capture the parameter uncertainty in the model by randomly sampling from parameter distributions of the input parameters. The red square indicates the point at which the mean cost and mean QALY values intersect.

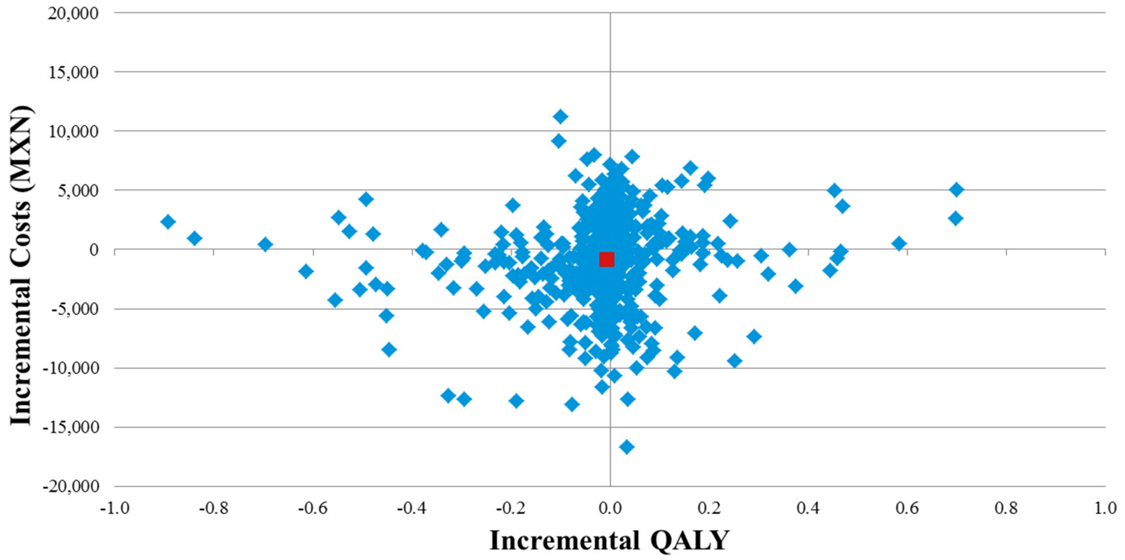

Figure S1. Cost-effectiveness plane for the outcome of the probabilistic sensitivity analysis (discounted).

Table S20 shows the PSA outcomes by quadrant. Southeast is the quadrant with the most model runs, where lpIF is less expensive and more effective.

Table S20. Allocation of PSA outcomes by quadrants of the cost-effectiveness plane.

Quadrant	Allocation
Northeast quadrant: lpIF is more expensive and more effective	20.1%
Northwest quadrant: lpIF is more expensive and less effective	16.0%
Southeast quadrant: lpIF is less expensive and more effective	32.7%
Southwest quadrant: lpIF is less expensive and less effective	31.2%

3. Validation

In order to assess the validity of the predictions of the model, Table S21 compares the result of the model specified for the current situation in Mexico (individual characteristics based on descriptive statistics for the Mexican population and using standard high-protein formula) with the observed outcomes for the Mexican population. It demonstrates that the model fairly accurately predicts the current life expectancy in Mexico and is the same ballpark for the other BMI and disease risk outcomes.

Table S21. Comparison of model predictions with the observed characteristics of the Mexican population [32].

Model Outcome	Model Prediction (Standard High Protein Formula)	Observed
Life years (LYs)	77.5 years	77 years
% diabetes	14.8%	17% to 21%
% angina	8.6%	4.8% to 9.2%
% MI	3.3%	
% stroke	0.27%	0.21%
% becoming obese (BMI ≥30)	17.1%	32.8%
Average lifetime BMI	28.2	31.3

Table S22 provides the individual characteristics considered in the model, broken down by age groups.

Table S22. Age-dependent individual characteristics.

Age Range	Males (mg/dL)	Standard Error	Female (mg/dL)	Standard Error	Source
**Fasting Glucose Level (FGL)**					
[18, 29]	88.7	4.53	80.9	4.13	[34]
[30, 39]	90.3	4.61	89.1	4.55	
[40, 49]	90.1	11.60	87.2	10.70	
[50, 59]	90.1	11.60	87.2	10.70	
[60, 69]	90.1	11.60	87.2	10.70	
[70, 79]	117.4	5.99	109.2	5.57	
[80, 100]	96.4	4.92	89.5	4.57	
[101, 199]	96.4	4.92	89.5	4.57	
**Systolic Blood Pressure (SBP)**					
[18, 29]	120.40	6.14	113.80	5.81	[34]
[30, 39]	122.30	6.24	116.90	5.96	
[40, 49]	124.90	6.37	123.10	6.28	
[50, 59]	128.30	6.55	129.00	6.58	
[60, 69]	132.50	6.76	133.60	6.82	
[70, 79]	134.70	6.87	137.50	7.02	
[80, 100]	132.90	6.78	137.70	7.03	
[101, 199]	132.90	6.78	137.70	7.03	

Table S22. *Cont.*

Age Range	Males (mg/dL)	Standard Error	Female (mg/dL)	Standard Error	Source
		**High Density Lipoprotein (HDL)**			
[18, 29]	36.70	0.66	40.00	0.59	[35]
[30, 39]	36.70	0.71	39.40	0.66	
[40, 49]	38.40	0.87	39.90	0.64	
[50, 59]	37.80	1.02	43.30	1.02	
[60, 100]	36.20	0.94	43.00	1.25	
[101, 199]	36.20	0.94	43.00	1.25	
		**Cholesterol/HDL-ratio**			
[18, 29]	5.04	0.26	4.75	0.24	[35]
[30, 39]	5.12	0.26	5.02	0.26	
[40, 49]	5.35	0.27	5.10	0.26	
[50, 59]	5.32	0.27	5.26	0.27	
[60, 100]	5.49	0.28	5.09	0.26	
[101, 199]	5.49	0.28	5.09	0.26	
		**Smoking Status**			
[18, 29]	34.7%	1.77%	10.7%	0.55%	[34]
[30, 39]	33.1%	1.69%	9.7%	0.49%	
[40, 49]	30.9%	1.58%	11.1%	0.57%	
[50, 59]	28.9%	1.47%	10.1%	0.52%	
[60, 69]	25.3%	1.29%	5.1%	0.26%	
[70, 79]	15.9%	0.81%	6.2%	0.32%	
[80, 100]	11.0%	0.56%	2.8%	0.14%	
[101, 199]	34.7%	1.77%	10.7%	0.55%	

Values for ages 18 to 20 were unavailable and, thus, assumed to be the same as those for the age range of 20 to 29. The standard error is calculated based on the assumption that this variable is normally distributed with 95% of the area within 1.96 standard deviations of the mean.

References

1. Druet, C.; Stettler, N.; Sharp, S.; Simmons, R.K.; Cooper, C.; Smith, G.D.; Ekelund, U.; Lévy-Marchal, C.; Jarvelin, M.R.; Kuh, D.; *et al.* Prediction of childhood obesity by infancy weight gain: An individual-level meta-analysis. *Paediatr. Perinat. Epidemiol.* **2012**, *26*, 19–26.
2. Østbye, T.; Malhotra, R.; Landerman, L.R. Body mass trajectories through adulthood: Results from the National Longitudinal Survey of Youth 1979 Cohort (1981–2006). *Int. J. Epidemiol.* **2011**, *40*, 240–250.
3. World Health Organization. *Non-Communicable Diseases Indicators*. May 2015. Available online: https://apps.who.int/infobase/Indicators.aspx (accessed on 4 May 2015).
4. World Health Organization. Global Health Observatory Data Repository. Segmented Life Tables for Mexico, 2011. Available online: http://apps.who.int/gho/athena/data/GHO/LIFE_0000000029,LIFE_0000000030,LIFE_0000000031,LIFE_0000000032,LIFE_0000000033,LIFE_0000000034,LIFE_0000000035?filter=COUNTRY:NGA&format=xml&profile=excel (accessed on 4 May 2015).
5. Stern, M.P.; Williams, K.; Haffner, S.M. Identification of persons at high risk for type 2 diabetes mellitus: Do we need the oral glucose tolerance test? *Ann. Internal Med.* **2002**, *136*, 575–581.
6. Wilson, P.W.; Bozeman, S.R.; Burton, T.M.; Hoaglin, D.C.; Ben-Joseph, R.; Pashos, C.L. Prediction of first events of coronary heart disease and stroke with consideration of adiposity. *Circulation* **2008**, *118*, 124–130.
7. D'Agostino, R.B.; Russell, M.W.; Huse, D.M.; Ellison, R.C.; Silbershatz, H.; Wilson, P.W.; Hartz, S.C. Primary and subsequent coronary risk appraisal: New results from the Framingham study. *Am. Heart J.* **2000**, *139*, 272–281.
8. Bonaca, M.P.; Braunwald, E.; de Ferrari, G.M.; Isaza, D.; Lewis, B.S.; Mehrhof, F.; Merlini, P.A.; Murphy, S.A.; Sabatine, M.S.; *et al.* Vorapaxar for secondary prevention of thrombotic events for patients with previous myocardial infarction: A prespecified subgroup analysis of the TRA 2 P-TIMI 50 trial. *Lancet* **2012**, *380*, 1317–1324.

9. Wallentin, L.; Becker, R.C.; Budaj, A.; Cannon, C.P.; Emanuelsson, H.; Held, C.; Horrow, J.; Husted, S.; James, S.; Katus, H.; *et al.* Ticagrelor *versus* clopidogrel in patients with acute coronary syndromes. *N. Engl. J. Med.* **2009**, *361*, 1045–1057.
10. Diener, H.-C.; Bogousslavsky, J.; Brass, L.M.; Cimminiello, C.; Csiba, L.; Kaste, M.; Leys, D.; Matias-Guiu, J.; Rupprecht, H.J.; MATCH Investigators. Aspirin and clopidogrel compared with clopidogrel alone after recent ischaemic stroke or transient ischaemic attack in high-risk patients (MATCH): Randomised, double-blind, placebo-controlled trial. *Lancet* **2004**, *364*, 331–337.
11. Trueman, P.; Lowson, K.; Bending, M.; Ganderton, M; Chaplin, S; Wright, D; Duffy, S; Saxby, R.. Bowel Cancer Services: Costs and Benefits. Final Report to the Department of Health. Available online: https://www.shef.ac.uk/polopoly_fs/1.44049!/file/FinalBowelCancerSummaryReport-Apr07.pdf (accessed on 4 May 2015)
12. Ridker, P.M.; Manson, J.E.; Gaziano, J.M.; Buring, J.E.; Hennekens, C.H. Low-dose aspirin therapy for chronic stable angina: A randomized, placebo-controlled clinical trial. *Ann. Internal Med.* **1991**, *114*, 835–839.
13. Instituto Nacional de Estadistica y Geografia. Main Causes of Mortality Habitual Residence, Age and Sex of the Deceased, 2012 Available online: http://www.inegi.org.mx/est/contenidos/proyectos/registros/vitales/mortalidad/tabulados/PC.asp?t=14&c=11817 (accessed on 4 May 2015).
14. Hospital Episode Statistics. 2009/2010 Provisional Linked HES-ONS Mortality Data Aggregated by Diagnosis/Procedure, 2010. Available online: http://www.hscic.gov.uk/hesonsmortality (accessed on 4 May 2015).
15. Instituto Nacional de Estadistica y Geografia. National Consumer Price Index and its Components, 2014. Available online: http://www.inegi.org.mx/sistemas/IndicePrecios/Cuadro.aspx?nc=CA55&T=%C3%8Dndices (accessed on 4 May 2015).
16. Instituto Mexicano del seguro Social Grupos Relacionados con el Diagnostico (DRG)—Cedula Medico-Economica (CME), 2012. Available online: http://201.144.108.20/profesionales/Pages/grd-cme.aspx (accessed on 4 May 2015).
17. Reynales-Shigematsu, L.M.; Rodríguez-Bolaños Rde, L.; Jiménez, J.A.; Juárez-Márquez, S.A.; Castro-Ríos, A.; Hernández-Avila, M. Health care costs attributable to tobacco consumption on a national level in the Mexican Social Security Institute. *Salud Pública Méx*. **2006**, *48*, s48–s64.
18. Bolaños, R.; de los Ángeles, R.; Myriam, R.S.L.; Alberto, J.R.J.; Arturo, J.M.S.; Mauricio, H.Á. Costos directos de atención médica en pacientes con diabetes mellitus tipo 2 en México: Análisis de microcosteo. *Rev. Panam. Salud Publica* **2010**, *28*, 412–420.
19. Sullivan, P.W.; Slejko, J.F.; Sculpher, M.J.; Ghushchyan, V. Catalogue of EQ-5D scores for the United Kingdom. *Med. Dec. Making* **2011**, *31*, 800–804.
20. Hakim, Z.; Wolf, A.; Garrison, L.P. Estimating the effect of changes in body mass index on health state preferences. *Pharmacoeconomics* **2002**, *20*, 393–404.
21. Geier, A.B.; Foster, G.D.; Womble, L.G.; McLaughlin, J.; Borradaile, K.E.; Nachmani, J.; Sherman, S.; Kumanyika, S.; Shults, J. The relationship between relative weight and school attendance among elementary schoolchildren. *Obesity* **2007**, *15*, 2157–2161.
22. Organisation for Economic Co-Operation and Development OECD Family Database: 2. The Labour Market Position of Families (LMF). Available online: http://www.oecd.org/els/family/database.htm#labour_market (accessed on 4 May 2015).
23. Brink, E.; Brändström, Y.; Cliffordsson, C.; Herlitz, J.; Karlson, B.W. Illness consequences after myocardial infarction: Problems with physical functioning and return to work. *J. Adv. Nurs.* **2008**, *64*, 587–594.
24. Kersten, P.; George, S.; Low, J.; Ashburn, A.; McLellan, L. The Subjective Index of Physical and Social Outcome: Its usefulness in a younger stroke population. *Int. J. Rehabil. Res.* **2004**. 27, 59–63.
25. Froelicher, E.; Kee, L.L.; Newton, K.M.; Lindskog, B.; Livingston, M. Return to work, sexual activity, and other activities after acute myocardial infarction. *Heart Lung* **1993**, *23*, 423–435.
26. Daniel, K.; Wolfe, C.D.; Busch, M.A.; McKevitt, C. What are the social consequences of stroke for working-aged adults? A systematic review. *Stroke* **2009**, *40*, e431–e440.
27. Ploug, U.J. The Cost of Diabetes-Related Complications: Registry-Based Analysis of Days Absent from Work. *Econ. Res. Int.* **2013**, doi:10.1155/2013/618039.
28. Brown, R.; Kendall, M.; Halpern, M. Cost Analysis of Once-Daily ISMN versus twice-daily ISMN or transdermal patch for nitrate prophylaxis. *J. Clin. Pharmacy Ther.* **1997**, *22*, 67–76.

29. Organisation for Economic Co-operation and Development. OECD Employment Outlook. Available online: http://www.oecd.org/els/emp/oecdemploymentoutlook.htm (accessed on 4 May 2015).
30. Hakkaart-van Rooijen, L.; Tan, S.; Bouwmans, C. *Dutch Costing Manual*; Institute for Medical Technology Assessment (By order of the Board of Health Insurances): Rotterdam, The Netherlands, 2010.
31. Chartered Institute of Personnel and Development. *Recruitment, Retention and Turnover. Annual Survey Report 2007*; Chartered Institute of Personnel and Development: London, UK, 2007.
32. World Health Organization. Global Health Observatory. Available online: http://www.who.int/gho/publications/world_health_statistics/en/ (accessed on 4 May 2015).
33. Ekelund, U.; Ong, K.; Linne, Y.; Neovius, M.; Brage, S.; Dunger, D.B.; Wareham, N.J.; Rössner, S. Upward weight percentile crossing in infancy and early childhood independently predicts fat mass in young adults: The Stockholm Weight Development Study (SWEDES). *Am. J. Clin. Nutr.* **2006**, *83*, 324–330.
34. World Health Organization. WHO Global Infobase. Available online: https://apps.who.int/infobase/Indicators.aspx (accessed on 4 May 2015).
35. Aguilar-Salinas, C.A.; Gómez-Pérez, F.J.; Rull, J.; Villalpando, S.; Barquera, S.; Rojas, R. Prevalence of dyslipidemias in the Mexican National Health and Nutrition Survey 2006. *Salud Pública Méx.* **2010**, *52* (Suppl. 1), S44–S53.

© 2016 by the authors; licensee MDPI, Basel, Switzerland. This article is an open access article distributed under the terms and conditions of the Creative Commons by Attribution (CC-BY) license (http://creativecommons.org/licenses/by/4.0/).
